# CYP3A4 Mutation Causes Vitamin D-Dependent Rickets Type 3: A Case Report in Saudi Arabia

**DOI:** 10.7759/cureus.49976

**Published:** 2023-12-05

**Authors:** Abdullah Al-Ashwal, Asma Al Zahrani, Nada Dammas, Lujain Aletani, Raghad Alhuthil

**Affiliations:** 1 Pediatric Endocrinology, King Faisal Specialist Hospital and Research Centre, Riyadh, SAU; 2 Pediatrics, King Faisal Specialist Hospital and Research Centre, Riyadh, SAU

**Keywords:** type 3, vitamin d deficiency, rickets, hereditary, cyp3a4, vddr3

## Abstract

Rickets is a childhood disorder of vitamin D deficiency that is characterized by growth retardation and impairment in skeletal mineralization. Vitamin D deficiency is usually due to decreased dietary vitamin D intake, decreased sunlight exposure, or genetic defects. A recurrent gain-of-function missense mutation (p.I301T) in the gene encoding *CYP3A4* has been identified as a cause of excessive inactivation of vitamin D metabolites that causes vitamin D-dependent rickets type 3 (VDDR3). We hereby report a case of a six-year-old girl with poor growth and bone deformities, such as genu valgum. In addition, the patient has a strong family history of short stature and bone deformities. She continues to receive multidisciplinary care, and the finding of a heterozygous missense variant in *CYP3A4*: c.902 T > C; p.Ile301Thr in the *CYP3A4* gene confirms the diagnosis of VDDR3. To our knowledge, this is the first case to be reported in Saudi Arabia and the fourth case in the literature. Our findings highlight the importance of vitamin D in those with high activity in *CYP3A4* to maintain vitamin D hemostasis, and we need to reach optimal doses to help them maintain their biochemical and radiological finding within the normal range.

## Introduction

Rickets is a childhood disorder of vitamin D deficiency that is characterized by growth retardation and impairment in skeletal mineralization. Vitamin D deficiency is usually due to decreased dietary intake of vitamin D, decreased sunlight exposure, or genetic defects [1].

There are genetic forms of rickets that have been identified. Despite patients living in geographical locations with an abundance of sunlight, these forms are known as vitamin D-dependent rickets (VDDRs). They are characterized by early-onset rickets due to mutations in genes that encode proteins required for vitamin D homeostasis or responsiveness of target tissue [2].

In order for vitamin D to become fully active, it undergoes two enzymatic conversions to the active form, which is 1,25-dihydroxyvitamin D. However, VDDRs have three broad classifications. In VDDR type 1 (VDDR1), there are mutations in the gene encoding either the renal 1-α hydroxylase (*CYP27B1*: VDDR-1A) or the hepatic 25-hydroxylase (*CYP2R1*: VDDR-1B) [2,3,4] On the other hand, VDDR type 2 (VDDR2) is due to defects in vitamin D receptor signaling, which occurs as a result of mutations in the gene encoding vitamin D receptor (*VDR*: VDDR-2A) or heterogeneous nuclear ribonucleoprotein C (*HNRNPC*: VDDR-2B) [5,6], a VDR coactivator. A recurrent gain-of-function missense mutation (p.I301T) in the gene encoding CYP3A4 has been identified as a cause of excessive inactivation of vitamin D metabolites and that cause VDDR type 3 (VDDR3) [2].

In this paper, we report a case of a six-year-old girl with poor growth and bone deformities, who clinically met the diagnostic requirement of VDDR3, which was newly reported by Roizen et al. (2018). VDDR3 is reported to be caused by a mutation in *CYP3A4* and leads to vitamin D deficiency by accelerated vitamin D metabolite inactivation [7] in two unrelated subjects with early-onset rickets who were poorly responsive to standard treatment regimens. Recently, Mantoanelli et al. in 2023 published a third case of VDDR3 due to the same *CYP3A4* mutation in a young boy who presented with bone deformities associated with poor growth [8].

## Case presentation

A six-year-old Saudi girl with genu valgum was referred to pediatric endocrinology service for further management. She was born at full-term normal pregnancy to consanguineous parents. Her birth weight and length were normal, and she had no post-natal complications.

Earlier at the age of two years, the family noted she had a delay in speech and walking. When she eventually started to walk at the age of three years, the family noticed that she had knock knees and an unsteady gait. She has a strong family history of rickets. Her parents are first cousins; the father has a history of rickets, short stature, and genu varum, corrected surgically and followed with orthopedics, while the mother is healthy. She only has one sister who is healthy with no bone deformities or similar presentation. There was no other family history of rickets or metabolic bone disease.

The patient was diagnosed with nutritional rickets. Based on laboratory investigations at the referring hospital, she had low calcium of 1.78 mmol/L (normal: 2.1-2.6), normal phosphate of 1 mmol/l (normal: 1.2-2), and high alkaline phosphatase of 423 IU/L (normal: 142-335) with high parathormone (PTH) around 200 pg/ml (normal: 15-65). However, 25 vitamin D was low, i.e., 5.3 ng/dl (normal >75). She has a normal liver and renal function tests. She was started on vitamin D and calcium supplements, and the family noticed mild improvement. One year later, her labs in the local hospital showed calcium of 2.44 mmol/L, Po4 of 1.5 mmol/L, 25 hydroxyvitamin D of 19.32 nmol/L, and PTH of 116.70 pg/ml despite vitamin D supplementation.

**Figure 1:**
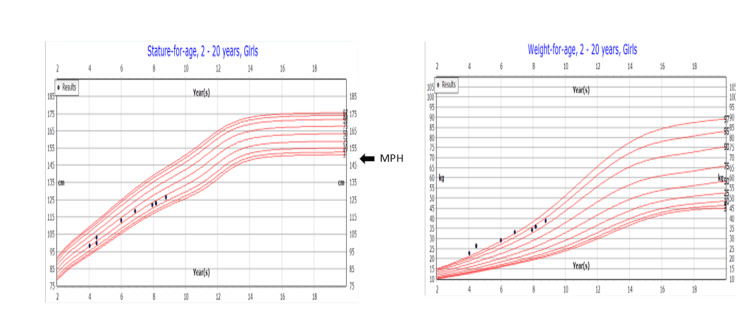
Centers for Disease Control and Prevention (CDC) chart for our patient weight and height, with methylphenidate (MPH) of 147 cm‎±10.

The patient presented to our clinic at the age of five years. Her growth parameters showed a height of 113 cm at the 39th percentile (-0.25 standard deviation (SD)), and her weight was 29.3 kg at 97.64 centiles (1.98 SD). Her methylphenidate (MPH) was 147‎±10 cm (Figure 1). Her physical examination revealed no dysmorphic features. She had mild frontal bossing and no widening in the wrists, and her gait was normal with mild genu valgum. 

**Figure 2:**
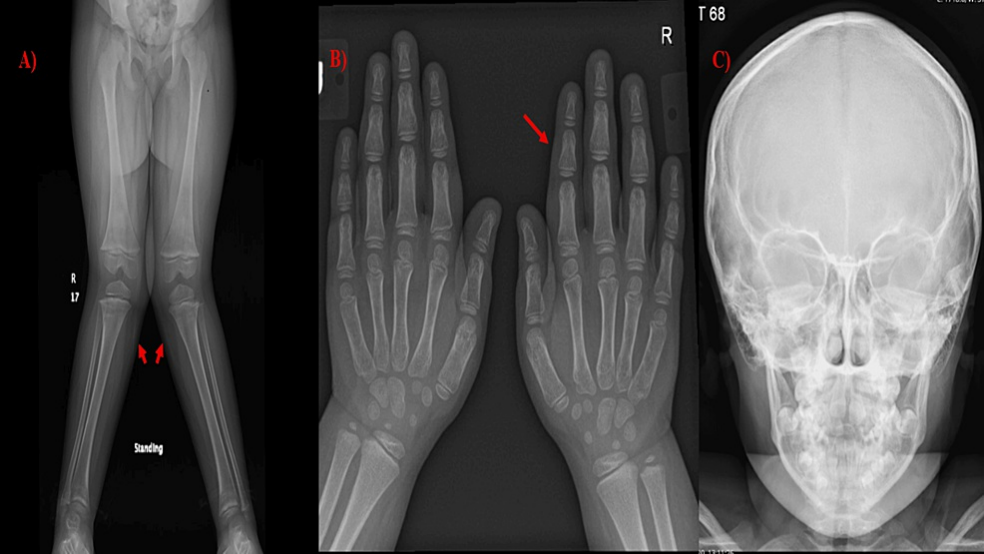
Skeletal survey showing moderate bilateral genu/tibia valgus deformities (bilateral genu valgus deformity). The right genu valgus deformity angle measurement is 28.1 degrees. The left genu valgus deformity angle measurement is 33.7 degrees (red arrow) (image A). In addition, mild osteopenia was present (image B). However, the patient had a normal dysmorphic skeletal survey with no evident dysmorphic skeletal features (image C).

Her initial biochemical tests showed normal levels of calcium (2.28 mmol/L), phosphate (1.2 mmol/L), alkaline phosphatase (234 IU/l), high PTH (79.2 ng/L), 25-hydroxyvitamin D (32 nmol/L), and 1,25-dihydroxyvitamin D (82 ng/L). She was on alfacalcidol 0.5 mcg daily. In addition, a radiograph of the lower limbs revealed a bilateral genu valgus deformity (Figure 2).

Given that the patient has a strong family history of rickets with consanguineous parents, a whole skeletal dysplasia panel was sent, which yielded inconclusive results. Subsequently, whole exome sequencing (WES) was conducted after the conclusion of the clinical investigations, where a heterozygous missense variant in CYP3A4: c.902 T > C; p.Ile301Thr in the CYP3A4 gene was discovered that is known to cause an abnormal bone mineralization process and autosomal dominant VDDR3.

**Figure 3:**
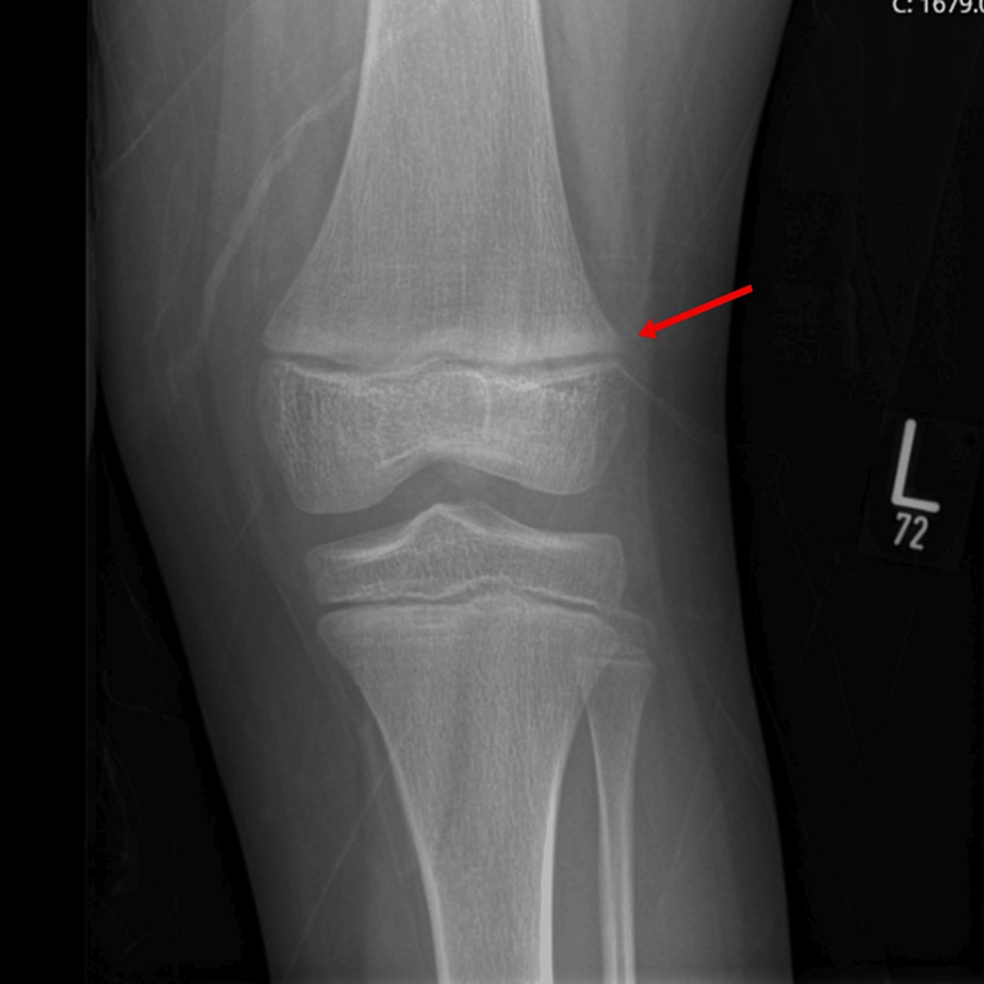
Faint metaphyseal sclerosis involving distal femurs (red arrow), suggestive of treated rickets.

Upon follow-up at the age of eight years, the patient continued to receive alfacalcidol at a dosage of 1.5-2 mcg, along with a daily calcium supplement of 30 mg/kg. The patient clinically improved (mild genu valgum), and her height was -0.87 SD. Her repeated labs are shown in Table 1. However, the skeletal survey showed generalized osteopenia with a faint metaphyseal sclerosis involving bilateral distal femurs, suggestive of treated rickets (Figure 3). Currently, the patient was started on a weekly dose of vitamin D 50,000 IU, and alfacalcidol was discontinued.

**Table 1:** Clinical data during follow-up SDS: standard deviation score, PTH: parathormone, ALP: alkaline phosphatase, ND: not done, H: high, L: low, N: normal

Age in years (treatment), during follow-up	Dose	Height cm (SDS)	Weight kg (SDS)	Calcium mmol/L (N: 2.1 - 2.6)	PTH pg/mL (N: 15 - 65)	ALP IU/L (N: 142 - 335)	25-hydroxy vitamin D nmol/L (N: >75)	1,25-Dihydroxyvitamin d ng/L (N: 20 - 80)	1,25-Dihydroxyvitamin D ng/L (N: 20 - 80)
At 3 years	-	ND	ND	1.78 (L)	200 (H)	423 (H)	5.3 (L)	ND	ND
At 3 years (3 months after cholecalciferol treatment)	1000-2000 IU	ND	ND	2.44 (N)	116.7 (H)	250 (N)	19.32 (L)	ND	ND
At 5 years (alfacalcidol)	4 mcg/day	113 (-0.25)	29.3 (1.98)	2.28 (N)	79.2 (H)	234 (N)	32 (L)	82 (H)	82 (H)
At 7 years (alfacalcidol)	3-4 mcg/day	122 cm (-0.94)	34.2 kg (1.46)	2.22 (N)	214 (H)	234 (N)	34 (L)	ND	ND
At 8 years (alfacalcidol) (21/8/2023)	1.5-2 mcg/day	126 cm (-0.87)	38.3 kg (1.49)	2.22 (N)	351 (H)	330 (N)	20 (L)	ND	ND
3 months after a high dose of cholecalciferol	50000 IU weekly	-	-	2.35 (N)	-	234(N)	57(L)	ND	ND

Approximately three months after beginning cholecalciferol 50,000 IU weekly, her lab results showed a significant improvement. Her vitamin D level was elevated to 57, two days after taking the cholecalciferol dose, and her ALP was also reduced to 234.

## Discussion

This is the first case reported in Saudi Arabia with VDDR3. However, our case is the fourth case reported in the literature with VDDR3 with the same variant mutation heterozygous missense variant in *CYP3A4*: c.902 T > C; p.Ile301Thr in the *CYP3A4* as previously reported cases. This disorder was first described in two unrelated cases by Roizen et al. in 2018 [7] and then recently by Mantoanelli et al. in 2023, who published the third case. Unlike the previously reported cases in the literature [7,8], which were Denovo mutations, in contrast, our patient had a family history of rickets, which warranted further genetic testing.

The most abundant P450 enzyme in the liver is *CYP3A4*, which is also expressed in large amounts in the intestines. It has an important role in various compounds, such as drugs, steroids, xenobiotics, and carcinogens [7]. The most applicable to our case is that it can oxidize and inactivate the vitamin D metabolites 25-hydroxyvitamin D and 1,25-dihydroxy vitamin D [9]. An example is demonstrated in patients who are taking anticonvulsants, where a common side effect is vitamin D deficiency [9]. In addition, polymorphism in *CYP3A4* is associated with loss of bone density [10]. Unlike the previously reported cases in the literature [7,8], our patient had a family history of rickets, which warranted further genetic testing. As previously mentioned, VDDR1 and VDDR2 are caused by either defects in the synthesis of vitamin D metabolites or responsiveness to 1,25-dihydroxy vitamin D. VDDR3, on the other hand, is caused by accelerated inactivation of vitamin D metabolites. In contrast to types 1 and 2, which are inherited in an autosomal recessive fashion, VDDR3 is inherited in an autosomal dominant fashion [7].

The clinical findings and presentation in our case are consistent with previous literature findings, including clinical and biochemical findings of nutritional rickets. Nutritional rickets are the most common cause of childhood rickets. However, genetic forms of rickets should still be considered in the differential diagnosis of vitamin D deficiency, including accelerated vitamin D inactivation as a risk factor for vitamin D deficiency that can cause VDDR3 especially If not responding to conventional doses of vitamin D [7].

Finally, our patient had mild improvement in her growth, and bone deformities were achieved after treatment with cholecalciferol conventional doses (alfacalcidol). By contrast, her biochemical labs still fluctuated. This proves the importance of early identification and diagnosis of this disorder. Our findings highlight the importance of vitamin D in those who have high activity in *CYP3A4* to maintain vitamin D hemostasis, and we need to reach optimal doses to help them to maintain their biochemical and radiological findings within the normal ranges.

## Conclusions

Rickets is a childhood disorder of vitamin D deficiency that is characterized by growth retardation and impairment in skeletal mineralization. Vitamin D deficiency is usually caused due to nutritional insufficiency or genetic defects. VDDR3 is a rare disorder, and only three cases have been reported in previous literature. The clinical and biochemical findings are similar to those of nutritional rickets, which can cause the prevalence of VDDR3 to be underestimated. Our findings align with reported cases in the literature, emphasizing the necessity to consider genetic testing for this type of rickets that shows resistance to conventional therapy and exhibits genetic anomalies. The optimal dose of cholecalciferol for treatment remains to be determined. However, in our case, a weekly dose of 50,000 IU was sufficient to normalize laboratory parameters. Nevertheless, the optimal high dose of cholecalciferol for treating VDDRs still needs further investigation and monitoring to reach a consensus on the age-dependent optimal dose for treating children and preventing growth stunting.

## References

[1] Terushkin V, Bender A, Psaty EL, Engelsen O, Wang SQ, Halpern AC (2010). Estimated equivalency of vitamin D production from natural sun exposure versus oral vitamin D supplementation across seasons at two US latitudes. J Am Acad Dermatol.

[2] Kitanaka S, Takeyama K, Murayama A (1998). Inactivating mutations in the 25-hydroxyvitamin D3 1alpha-hydroxylase gene in patients with pseudovitamin D-deficiency rickets. N Engl J Med.

[3] Cheng JB, Levine MA, Bell NH, Mangelsdorf DJ, Russell DW (2004). Genetic evidence that the human CYP2R1 enzyme is a key vitamin D 25-hydroxylase. Proc Natl Acad Sci U S A.

[4] Thacher TD, Fischer PR, Singh RJ, Roizen J, Levine MA (2015). CYP2R1 mutations impair generation of 25-hydroxyvitamin D and cause an atypical form of vitamin D deficiency. J Clin Endocrinol Metab.

[5] Chen H, Hewison M, Hu B, Adams JS (2003). Heterogeneous nuclear ribonucleoprotein (hnRNP) binding to hormone response elements: a cause of vitamin D resistance. Proc Natl Acad Sci U S A.

[6] Pike JW, Dokoh S, Haussler MR, Liberman UA, Marx SJ, Eil C (1984). Vitamin D3--resistant fibroblasts have immunoassayable 1,25-dihydroxyvitamin D3 receptors. Science.

[7] Roizen JD, Li D, O'Lear L (2018). CYP3A4 mutation causes vitamin D-dependent rickets type 3. J Clin Invest.

[8] Mantoanelli L, de Almeida CM, Coelho MC, Coutinho M, Levine MA, Collett-Solberg PF, Bordallo AP (2023). Vitamin D-dependent rickets type 3: a case report and systematic review. Calcif Tissue Int.

[9] Wang Z, Senn T, Kalhorn T (2011). Simultaneous measurement of plasma vitamin D(3) metabolites, including 4β,25-dihydroxyvitamin D(3), using liquid chromatography-tandem mass spectrometry. Anal Biochem.

[10] Kang YS, Park SY, Yim CH (2009). The CYP3A4*18 genotype in the cytochrome P450 3A4 gene, a rapid metabolizer of sex steroids, is associated with low bone mineral density. Clin Pharmacol Ther.

